# The Effect of Telerehabilitation on Missed Appointment Rates

**DOI:** 10.5195/ijt.2018.6258

**Published:** 2018-12-11

**Authors:** LYN T. COVERT, JOHN T. SLEVIN, JESSICA HATTERMAN

**Affiliations:** 1 DEPARTMENT OF VETERANS AFFAIRS MEDICAL CENTER, LEXINGTON, KY, USA; 2 UNIVERSITY OF KENTUCKY, LEXINGTON, KY, USA; 3 DEPARTMENT OF NEUROLOGY, COLLEGE OF MEDICINE, UNIVERSITY OF KENTUCKY, LEXINGTON, KY

**Keywords:** Lee Silverman Voice Treatment (LSVT®), No-show, Missed appointments, Telerehabilitation, Voice therapy

## Abstract

The purpose of this study was to examine the effect of telerehabilitation on missed appointment rates in a rehabilitation clinic. Clients fail to attend scheduled appointments for a variety of reasons. Unmet appointments represent a loss of financial support as well as diminished efficiency and capacity to provide services. Speech therapy utilizing multiple appointments is most difficult to maintain during a treatment regimen. This may cause individuals to miss appointments and therefore not achieve desired results. For this study, researchers utilized an intense speech therapy technique, the Lee Silverman Voice Treatment (LSVT®) to measure compliance with scheduled appointments. Participants were randomized to either in-person treatment or telerehabilitation treatment at a site distant from the speech-language pathologist. Participants in the telerehabilitation (TR) condition completed significantly more appointments than participants in the in-person (IP) condition. When comparing results of treatment for each condition, there were no significant differences in outcome whether treated in the IP or TR condition of the study for monologue and picture description tasks, which are closely associated with conversational speech. There was a difference in the reading task with participants demonstrating significantly better post treatment results in the IP condition. The reason for this disparity is unclear and warrants further study.

Missed appointments can create financial, capacity, and continuity issues in rehabilitation clinics. The financial loss attributed to missed appointments can be very high (Berg, et al., 2013). There are multiple factors leading to a missed appointment. Clients may fail to attend their appointments, commonly called no-shows, without prior notice, resulting in wasted capacity and a lost provider revenue opportunity. No-shows also include patients who cancel appointments on short notice (i.e., <24 hours). This equates to a no-show because a minimum time threshold is required to prepare for the procedure, yielding short notice rescheduling impractical. United States studies describe no-show rates in community practices that range from 5%–55% (Berg et al., 2013).

The occurrence of no-shows effectively increases the costs to a facility, which may create a patient access barrier (Berg et al., 2013). For example, missed appointments compromise continuity and quality of care for both the patients who do not show for their appointments and others who could have been scheduled in those appointment slots (DuMontier, Rindfleisch, Pruszynski, & Frey, 2013). Unfilled appointments represent a loss of financial support as well as diminished efficiency and capacity to provide services.

Patients provided several reasons for not attending appointments, in addition to merely forgetting them. Logistical issues included trouble getting off work, childcare, and transportation. Travel costs associated with driving to an appointment, taking time off from work, and other expenses are cost prohibitive to many individuals aging with a disability (Tindall & Huebner, 2009). Further, patients who feel better and those who feel too ill to travel fail to keep appointments. Therefore, a single issue does not determine if patients will present to a clinic for their scheduled appointments. If the use of telerehabilitation by a speech-language pathologist (SLP) can influence missed appointments resulting in added revenue and capacity and deliver the same quality of services to clients, it can represent an added return on investment (ROI). In the current atmosphere of healthcare reimbursement, an additional concern is providing care that is cost efficient to both client and facility. One way to examine cost effectiveness to facilities is to study the impact of telerehabilitation on missed appointments. The main purpose of this study was to compare missed appointment rates between the telerehabilitation and in-person conditions to determine if telerehabilitation could improve attendance in a rehabilitation clinic.

## TELEREHABILITATION AND SPEECH-LANGUAGE PATHOLOGY

Distance, insufficient resources, and mobility challenges may impede access to evidence-based treatment (Theodoros, Hill & Russell, 2016). Aging with a disabling condition significantly limits an individual’s activities of daily living and quality of life. Although multiple effective treatments are available to reduce the effects of chronic diseases, many individuals experience barriers, such as mobility deficits and transportation difficulties in accessing these treatments. Even younger individuals experience limited resources in accessing healthcare. Telerehabilitation may be a method of service delivery that could potentially eliminate or minimize barriers to accessing health care (Tindall, Huebner, Stemple & Kleinert, 2008). The American Telemedicine Association (ATA) defines telerehabilitation as the delivery of services via telecommunication technology (American Telemedicine Association, 2017).

Speech-language pathology services lend themselves to these types of applications. The use of telerehabilitation is a reliable and valid assessment and treatment tool to treat communication disorders (Kully, 2000; Lemaire, Boudrias & Greene, 2001; Scheideman-Miller, Clark, Smeltzer, Cloud, Carpenter, Hodge, et al., 2002; Sicotte, Lehoux, Fortier-Blanc & Leblanc, 2003;Georgeadis, Brennan, Barker, & Baron, 2004; Hill, Theodoros, Russell, Cahill, Ward & Clark, 2006; Keck & Doarn, 2014). Clients and SLPs are highly satisfied with telehealth delivery of services and outcome measures related to improvements in speech are positive. Therefore, speech-language pathology services appear to be well suited for telerehabilitation delivery as clients can see and hear a clinician give instructions and reciprocally the clinician can see and hear the clients’ responses, similar to a traditional in-person clinical setting.

## IDIOPATHIC PARKINSON’S DISEASE

This study targeted individuals diagnosed with Idiopathic Parkinson’s disease (IPD). The annualized age and gender-adjusted incidence rate of IPD is 13.4 per 100,000 rapidly increasing over the age of 60 years. The incidence rate for men is 91% higher than for women. The age and gender-adjusted rate is highest among Hispanics, followed by non-Hispanic whites, Asians, and Blacks (Van Den Eeden et al., 2003). Age is the most consistent risk factor, and with the increasing age of the veteran population (approximately 35–40% of the veterans are over age 65), the prevalence of IPD is predicted to be steadily rising in the future. Further, neurodegenerative diseases *in toto* are expected to surpass cancer as the second leading cause of death among elders by the year 2040. Although there is no cure, early detection and treatment can alter progression of IPD and enhance quality of life (Paulson & Stern, 1997).

IPD is a neurodegenerative disorder characterized by rigidity of striated muscles, causing difficulties in respiration, facial expression, swallowing, mastication, and speech. Therefore, individuals with IPD usually develop a speech disorder characterized by reduced loudness, hoarse and breathy voice, monotony of pitch, short rushes of speech, and imprecise consonants (Critchley, 1981; Darley, Aronson, & Brown, 1969a, 1969b). The inability to effectively communicate impairs the ability of patients with IPD to function in society and impacts their quality of life. A successful program developed to improve speech in these individuals is the Lee Silverman Voice Treatment (LSVT®) (Ramig, Countryman, Thompson & Horii, 1995; Ramig, Sapir, Fox, & Countryman, 2001). LSVT® was one of the earliest successful programs applied to treat the speech deficit of reduced loudness of IPD.

## LEE SILVERMAN VOICE THERAPY

LSVT® has demonstrated short and long-term (2-year) retention in loudness as well as generalized improvements in articulation, facial expression, swallowing, and communicative gesturing (Ramig et al., 2001). If the speech disorder associated with IPD can be corrected it may result in an improved quality of life for individuals living with this chronic disease, an additional benefit of this program. However, LSVT® treatment requires intense daily therapy for 4 weeks, a regimen that is difficult for many elderly veterans living in rural areas.

Massed practice as prescribed by LSVT® is consistent with principles of neuroplasticity, motor learning, skill acquisition, and muscle training (Fox, Ebersbach, Ramig, & Shapir, 2012). LSVT® Programs include: (1) an exclusive target to increase amplitude (loudness), (2) focus on sensory recalibration to help patients recognize movements with increased amplitude, and (3) training for self-cuing to facilitate long-term maintenance of treatment. Nonetheless, the high intensity and required consistency that make this program successful is also associated with a tendency for individuals to decline starting therapy or to miss therapy appointments. Frequency of treatment can be an obstacle to providing this therapy to clients due to mobility problems or employment constraints (Spielman, Ramig, Mahler, Halpern, & Gavin, 2007).

Some SLPs may withhold treatment or offer fewer weekly sessions to accommodate clients’ schedules. Although such treatment variations may be more convenient for clients, the effects of these modifications remain inconclusive (Stroud & Belin, 2004: Wohlert, 2004). The purpose of this study was to determine outcomes of one such modification: providing individuals with IPD an option of using telerehabilitation to enable them to receive services in a setting closer to their homes.

## METHOD

### PARTICIPANTS

In order to generalize results from this study to the real world experience of travel to medical facilities, there were no limits placed on distance to the primary site of origin for participants. The main medical center is located in a metropolitan area with a population density of 1000/square mile (www.opendatanetwork.com). In addition to the major metropolitan area, this medical center also serves a rural Appalachian area in southeast Kentucky with a population density of less than 250 per square mile (https://commons.wikimedia.org). A primary problem in this part of the country is that it is remote, miles from major highways and plagued by substandard infrastructure.

We recruited individuals from the catchment area of a medical center that serves both the metropolitan and rural areas described. The neurologist on the research team screened individuals from the neurology clinic to determine if they met inclusion criteria for the study. SLPs associated with the study oversaw the informed consent process. We screened and enrolled forty-eight individuals. They ranged in age from 54 to 87 years and were diagnosed with IPD from 1 to 15 years.

Twelve individuals withdrew before finishing treatment; thus, 36 participants (34 males, 2 females) completed the study. They were randomized (1:1) to either the Telerehabilitation (TR) condition or In-Person (IP) condition. Table 1 contains a summary of demographic information for those who completed the study.

#### RANDOMIZATION PROCEDURE

SLPs divided participants into two conditions for randomization: those who lived within a 30-mile radius of the medical center (local) and those who lived beyond this radius (remote). Separate randomization plans for local and remote living conditions of participants were generated using online randomization. Thus, participants in the local condition were randomized to either TR or in-person (IP) and those in the remote condition were also randomized to TR or IP. Participants characterized as local and randomized to TR received TR speech therapy in an adjoining room at the medical center. The reason for this type of randomization was to obtain a more accurate measure of missed appointments in addition to controlling for selection bias.

SLPs associated with the study performed routine voice assessments on all participants. They also completed the speech motor examination section of the Unified Parkinson’s Disease Rating Scale (UPDRS) (Fahn, Marsden, Goldstein & Caline, 1987). The UPDRS is a rating tool to follow the longitudinal course of Parkinson’s disease with 0= no disability to 4= severe disability. Table 2 presents descriptions of each score.

### PROCEDURES

The pretest and posttest assessments and procedures used by Ramig et al., (2001) were replicated and provided by LSVT® trained and certified SLPs. Participants completed recordings of vocal intensity measured in decibels (dB) of a sustained vowel, reading passage, monologue, and picture description were obtained in-person in the speech clinic both prior to and at the conclusion of treatment. During each probe task, sound pressure levels in dB were recorded using the LSVT® Companion ® System. This system includes an interactive patient interface and a calibrated microphone.

#### TRADITIONAL IN-PERSON CONDITION

The LSVT® approach prescribes three treatment tasks per therapy session to improve vocal intensity. These tasks include: (1) *maximum duration of a sustained vowel by the participant* to improve glottal competence and respiratory/laryngeal coordination; (2) *practice of pitch range* to improve range of motion of the cricothyroid muscle; and (3) *practice of maximum functional speech loudness drill* to increase phonatory effort (Ramig, et al. 1995).

LSVT® prescribes 16 therapy sessions over a 4-week period to complete the program. Participants completed homework assignments each day during treatment. Homework assignments included performing the same tasks used during treatment but with fewer repetitions. Participants completed one homework page each day while participating in the study.

Within one week of completing four weeks of treatment, participants returned to the speech clinic to undergo the post-treatment data collections previously described. SLPs performed sound pressure level calculation in the same manner described during baseline data collection.

#### TELEREHABILITATION CONDITION

Participants assigned to the telerehabilitation arm of this study received the identical therapy described previously, except they received therapy via telerehabilitation at an outpatient clinic close to their home.

#### TELEREHABILITATION TECHNOLOGY

Tandberg Profile 3000 MXP equipment was employed at each community based outpatient clinic (CBOC). The unit resembles a wide screen television monitor. Participants sat facing the monitor to begin voice therapy. Technicians at each CBOC manipulated the equipment. The technicians were trained nursing assistants employed at each CBOC.

Tandberg Centric 1700 MXP equipment was used at the main medical center. These are desktop units, similar to a 32-inch television monitor. Subjects randomized to the speech clinic for the telerehabilitation arm sat facing these monitors. SLPs used an Internet Protocol (IP) videoconference connection to access all telerehabilitation equipment. Each device had an Internet or Intranet IP address that enabled the systems to communicate with each other.

### STATISTICAL ANALYSIS

We implemented a Welch Two Sample t-test to examine pretest to posttest changes in measures of decibels (dB) for vowel prolongation, reading passage, picture description, and monologue, using the means to investigate improvements in vocal loudness in two delivery conditions with an alpha level of .05 for this test statistic.

## RESULTS

Thirty-six participants comprising 18 per condition completed this study. Participants in the TR condition completed an average number of 13.27 sessions. Participants in the IP completed an average of 10.5 therapy sessions. This difference was significant (t(30.2)= −1.99, p<0.03). Table 3 shows results.

Table 4 displays the pre- and post-LSVT® values for TR and IP conditions, standard deviations, changes, and *p* values of acoustic measures. Participants in the TR condition demonstrated significant improvements in dB for all tasks. In the IP condition, significant changes were observed with all tasks except monologue. A Bonferroni correction was applied to control for the possibility of a Type 1 error (four tests).

A comparison of LSVT® intervention using TR was at least equally effective to IP rehabilitation, with exception of the reading task. Results of this analysis are presented in Table 5 indicating that change in intensity levels of prolonged vowel (t(31.5)=0.84, p<0.4), picture description (t(33.7)=1.35, p<0.18), and monologue (t(33.6)=1.37, p<0.17 were not statistically different. These results allow us to reject the null hypothesis for vowel prolongation, picture description, and monologue indicating no differences in outcomes of the two methods of treatment delivery. It is unclear why post-LSVT® values for reading were significantly different between the two conditions. There may be confounding variables not yet identified. The researchers may undertake further exploration of this finding.

## DISCUSSION

Missed appointments cause a loss of financial resources leading to diminished efficiency and capacity to provide services. Medical center and clinic administrators are exploring ways to minimize this drain on health care resources. In this study, we explored telerehabilitation as a tool to decrease lost revenue and capacity by improving compliance in attendance in a rehabilitation setting. Decreasing missed appointments can ease financial costs of providing health care. Through improvements in managing healthcare resources, hospitals and clinics may increase their level of capacity to provide services to more individuals. In addition to minimizing lost revenue associated with missed appointments, telerehabilitation must also provide services equal to or better than in-person delivery of services.

Further, utilization of telerehabilitation technology can effect improvements for individuals with a voice disorder. Participants in both conditions achieved significant post treatment improvements in vocal intensity demonstrated by increased dB levels of vocal intensity of participants following voice therapy with the exception of monologue in the IP condition. When comparing results of treatment for each condition, there were no significant differences in outcome whether treated IP or TR condition of the study for monologue and picture description tasks, which are closely associated with conversational speech. There was a difference in the reading task with participants demonstrating significantly better post treatment results in the IP condition. The reason for this disparity is unclear and warrants further study.

The results support the hypothesis that participants in the TR condition completed more appointments than the IP condition. This difference was significant (t (30.2)= −1.99, p<0.03). The researchers conducted therapy in CBOCs close to participants’ homes for the TR condition. We did not consider distance from the main medical center when randomizing participants. Though outreach clinics were located closer to participants’ homes than the main medical center, some travel was involved. These findings suggest telerehabilitation can be a tool used to improve efficiency of rehabilitation clinics by enabling clients to have greater access to services. Future research should focus on in-home delivery of services using Internet Protocols (IP) to determine if elimination of travel can further compliance with therapy regimens.

## Figures and Tables

**Table 1:** Demographics of Participants

	TR				IP		

Gender	Age	Time P/O	UPDRS	Gender	Age	Time P/O	UPDRS
M	66	2	2	M	80	6	1
M	65	2	3	M	75	4	1
M	85	2	3	M	67	3	1
M	70	1	1	M	64	1	1
M	60	12	1	M	78	14	1
M	67	8	2	M	87	4	2
M	79	2	1	M	54	3	2
M	84	13	2	M	69	12	2
M	66	4	1	F	63	5	2
M	62	3	2	M	57	2	2
M	75	13	1	M	74	12	2
M	67	15	2	M	72	8	2
M	75	9	3	M	81	2	2
M	68	3	2	M	70	4	2
M	73	10	2	M	85	3	3
M	65	14	1	M	60	1	2
F	65	2	2	M	70	9	2
M	70	11	3	M	86	6	2

*Note*. TR=Telerehabilitation, IP=In-Person, P/O= post onset of diagnosis in years, UPDRS= Unified Parkinson’s Disease Rating Scale

**Table 2:** Unified Parkinson’s Disease Rating Scale (Fahn et al., 1987)

III. MOTOR EXAMINATION 18. Speech 0 = Normal 1 = Slight loss of expression, diction and/or volume 2 = Monotone, slurred but understandable; moderately impaired 3 = Marked impairment, difficult to understand. 4 = Unintelligible

**Table 3:** Mean Number of Therapy Sessions Completed

TR Condition	IP Condition	*t*	*df*	*P*
13.27	10.55	−1.99	30.17	0.027

*Note*. TR=Telerehabilitation, IP=In-Person

**Table 4:** Results of LSVT®

TR Condition						

Acoustic Parameters (dB)	Pre	Post	Change	*t*	*df*	*p*
Prolonged Vowel	71.2	80.56	9.4	6.9	17	<0.001
Reading Passage	66.68	70.05	3.6	5.6	17	<0.001
Monologue	64.72	67.91	3.1	6.4	17	<.001
Picture Description	66.61	69.31	2.7	4.2	17	<0.01

IP Condition						

Acoustic Parameters (dB)	Pre	Post	Change	*t*	*df*	*p*
Prolonged Vowel	72	81.73	9.8	6.4	17	<0.001
Reading Passage	67.2	72.82	5.7	3.3	17	<.05
Monologue	66.7	69.5	3.1	2.2	17	<.16
Picture Description	66.61	71.28	2.7	4.1	17	<0.01

*Note.* TR=Telerehabilitation, IP=In-Person, dB = decibels.

**Table 5:** Comparison of Results for Each Condition

	TR Condition			IP Condition					

Acoustic Parameters (dB)	Pre	Post	Change	Pre	Post	Change	*t*	*df*	*p*
Prolong Vowel	71.2	80.56	9.4	72	81.73	9.8	1.35	33.7	< 0.18
Reading Passage	66.68	70.05	3.6	67.2	72.82	5.7	1.99	32.5	< 0.05
Monologue	64.72	67.91	3.1	66.7	69.5	3.2	1.37	33.6	< 0.17
Picture Description	66.61	69.31	2.7	66.1	71.28	4.6	1.35	33.7	< 0.18

Note. TR=Telerehabilitation, IP=In-Person, dB = decibels.

## References

[b1-ijt-10-65] American Telemedicine Association (2017). Principles for delivering telerehabilitation services.

[b2-ijt-10-65] Berg BP, Murr M, Chermak D, Woodall J, Pignone M, Sandler RS, Denton BL (2013). Estimating the cost of no-shows and the effects of mitigation strategies. Medical Decision Making.

[b3-ijt-10-65] Critchley E (1981). Speech disorders of Parkinsonism: A review. Journal of Neurology, Neurosurgery & Psychiatry.

[b4-ijt-10-65] Darley F, Aronson A, Brown J (1969a). Differential diagnostic patterns of dysarthria. Journal of Speech and Hearing Research.

[b5-ijt-10-65] Darley F, Aronson A, Brown J (1969b). Clusters of deviant speech dimensions in the dysarthrias. Journal of Speech and Hearing Research.

[b6-ijt-10-65] DuMontier C, Rindfleisch K, Pruszynski J, Frey J (2013). A multi-method intervention to reduce no-shows in an urban residency clinic. Family Medicine.

[b7-ijt-10-65] Fahn S, Marsden G, Goldstein M, Caline D (1987). Recent developments in Parkinson’s disease.

[b8-ijt-10-65] Fox C, Ebersbach G, Ramig L, Sapir S (2012). LSVT® LOUD and LSVT® BIG: Behavioral treatment programs for speech and body movement in Parkinson disease. Parkinson’s Disease.

[b9-ijt-10-65] Georgeadis A, Brennan D, Barker L, Baron C (2004). Telerehabilitation and its effect on story retelling by adults with neurogenic communication disorders. Aphasiology.

[b10-ijt-10-65] Hill A, Theodoros D, Russell T, Cahill L, Ward E, Clark K (2006). An Internet-based telerehabilitation system for the assessment of motor speech disorders. A pilot study. American Journal of Speech-Language Pathology.

[b11-ijt-10-65] Keck C, Doarn C (2014). Telehealth technology applications in speech-language pathology. Telemedicine Journal & eHealth.

[b12-ijt-10-65] Kully D (2000). Telehealth in speech patholgoy: Applications to the treatment of stuttering. Journal of Telemedicine and Telecare.

[b13-ijt-10-65] Lemaire E, Boudrias Y, Greene G (2001). Low-bandwidth, Internet-based videoconferencing for physical rehabilitation consultations. Journal of Telemedicine and Telecare.

[b14-ijt-10-65] OpenDataNetwork (2016). https://www.opendatanetwork.com/entity/0400000US21/Kentucky/geographic.population.density?year=2016.

[b15-ijt-10-65] Paulson H, Stern MB, Watt R, Koller (1997). Clinical manifestations of Parkinson’s disease. Movement Disorders: Neurological Principles and Practice.

[b16-ijt-10-65] Ramig LO, Countryman S, Thompson LL, Horii Y (1995). Comparison of two forms of intensive speech treatment for Parkinson disease. Journal of Speech and Hearing Research.

[b17-ijt-10-65] Ramig L, Sapir S, Fox C, Countryman S (2001). Changes in vocal intensity following intensive voice treatment (LSVT®) in individuals with Parkinson disease: A comparison with untreated patients and normal age-matched controls. Movement Disorders.

[b18-ijt-10-65] Scheideman-Miller C, Clark P, Smeltzer S, Cloud A, Carpenter J, Hodge B, Prouty D (2002). Two year results of a pilot study delivering speech therapy to students in a rural Oklahoma school via telemedicine.

[b19-ijt-10-65] Sicotte C, Lehoux P, Fortier-Blanc J, Leblanc Y (2003). Feasibility and outcome evaluation of a telemedicine application in speech-language pathology. Journal of Telemedicine and Telecare.

[b20-ijt-10-65] Spielman J, Ramig L, Halpern A, Gavin W (2007). Effects of an extended version of Lee Silverman Voice Treatment on voice and speech in Parkinson’s disease. American Journal of Speech-Language Pathology.

[b21-ijt-10-65] Stroud A, Belin G (2004). Effects of intensive voice treatment (LSVT®) on ataxic and hypokinetic dysarthria: A condition case study.

[b22-ijt-10-65] Theodoros DG, Constantinescu G, Russell T, Ward EC, Wilson SJ, Wootton R (2009). Treating the speech disorder in Parkinson’s disease online. Journal of Telemedicine and Telecare.

[b23-ijt-10-65] Theodoros D, Hill A, Russell T (2016). Clinical and quality of life outcomes of speech treatment for Parkinson’s disease delivered to the home via telerehabilitation: A noninferiority randomized controlled trial. American Journal of Speech-Language Pathology.

[b24-ijt-10-65] Tindall L, Huebner R (2009). The impact of an application of telerehabilitation technology on caregiver burden. International Journal of Telerehabilitation.

[b25-ijt-10-65] Tindall L, Huebner R, Stemple J, Kleinert H (2008). Videophone-delivered voice therapy: A comparative analysis of outcomes to traditional delivery for adults with Parkinson’s disease. Telemedicine Journal & eHealth.

[b26-ijt-10-65] Van Den Eeden S, Tanner C, Bernstein A, Fross R, Leimpeter A, Bloch D, Nelson LM (2003). Incidence of Parkinson’s disease: Variation by age, gender, and race/ethnicity. American Journal of Epidemiology.

[b27-ijt-10-65] Wikimedia Commons (2010). https://commons.wikimedia.org/wiki/File:Kentucky_population_map.png.

[b28-ijt-10-65] Wohlert A (2004). Service delivery variables and outcomes of treatment for hypokinetic dysarthria in Parkinson’s disease. Movement Disorders.

